# Identification of Natural Killer Cell‐Associated Clusters in Skin Melanoma and the Impact on Prognosis and Drug Sensitivity

**DOI:** 10.1002/iid3.70143

**Published:** 2025-02-17

**Authors:** Jun Zhou, Renhui Cai, Danqun Zhang, Caifeng Chen

**Affiliations:** ^1^ Department of Dermatology Fuzhou University Affiliated Provincial Hospital, Fujian Provincial Hospital, Clinical Medical College of Fujian Medical University Fuzhou China

**Keywords:** drug sensitivity, immunotherapy, natural killer cell, skin melanoma, tumor microenvironment

## Abstract

**Background:**

Skin melanoma exhibits significant heterogeneity in clinical outcomes and treatment responses among patients. This study aimed to investigate natural killer (NK) cell clusters in skin melanoma, their impact on patient prognosis, and their value as biomarkers for tailoring treatment.

**Methods:**

We used data from TCGA, GSE19234, GSE65904, GSE244982, and GSE78220. A gene classifier was developed to identify two distinct clusters of melanoma patients. Survival analysis, NK cell infiltration levels, and responses to immune and targeted therapies were evaluated.

**Results:**

Unsupervised clustering revealed two distinct melanoma patient clusters with significant differences in NK cell activity and clinical outcomes. Cluster 1 showed higher NK cell infiltration, better overall survival (OS) (*p* < 0.0001), and greater activity in NK‐cell‐related pathways. In contrast, Cluster 2, characterized by lower NK cell activity and higher exhaustion markers, had poorer OS. Drug sensitivity analysis indicated that Cluster 1 was more responsive to most melanoma treatments, whereas Cluster 2 had higher sensitivity to trametinib (*p* < 0.001). The developed gene classifier had an AUC of 0.913 and effectively differentiated between clusters. Additionally, Cluster 1 showed better responses to immunotherapy with a higher rate of complete and partial responses (*p* < 0.001). These findings were validated in external databases.

**Conclusion:**

This study identifies two distinct NK‐cell‐related clusters in melanoma with differential prognoses and treatment responses. These findings underscore the importance of integrating NK‐cell‐related profiles into personalized treatment strategies, offering a pathway to optimize therapeutic outcomes based on specific immune profiles.

## Graphical abstract

Unsupervised clustering of NK cell‐related gene expression revealed two distinct patient clusters within TCGA‐SKCM data, each with significantly different clinical outcomes. Cluster 1 showed higher NK cell activity and better overall survival (OS), while Cluster 2 had lower NK cell levels and higher NK cell exhaustion, resulting in a poorer prognosis. Cluster 1 was more sensitive to most melanoma treatments, except trametinib, which showed better sensitivity in Cluster 2.

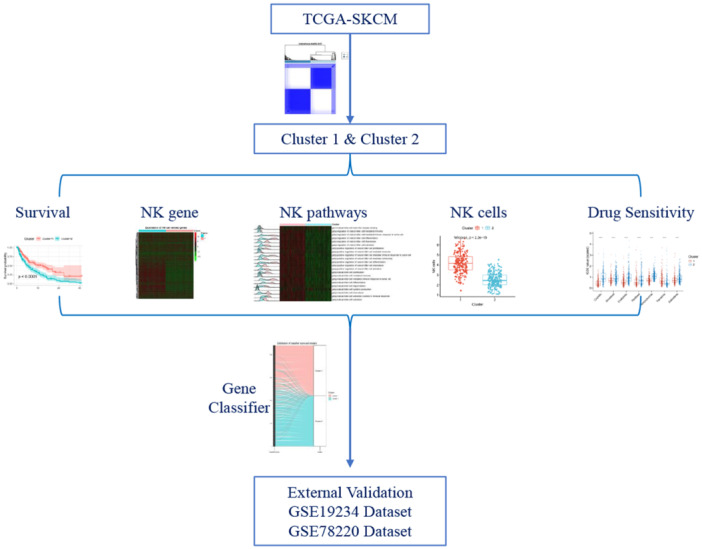

## 1 Introduction

Melanoma, a highly aggressive form of skin cancer, poses significant challenges due to its propensity for immune evasion and rapid progression. One of the critical factors contributing to melanoma's aggressiveness is its high mutational burden, which enhances its immunogenicity and influences the interaction between tumor cells and immune cells [1, 2]. Tumor cells in melanoma can rapidly adapt to the hostile tumor microenvironment (TME) by developing immune evasion mechanisms, including altering their antigenicity and releasing immune‐modulatory factors [3]. These mechanisms can influence the composition and activity of infiltrating immune cells, further facilitating tumor progression and metastasis [4].

Natural killer (NK) cells, key players in innate immunity, are crucial for their ability to recognize and destroy tumor cells without prior sensitization [5]. In melanoma, NK cells contribute significantly to the initial immune response against tumor cells [6, 7]. However, as the disease progresses, melanoma cells may develop strategies to evade NK‐cell‐mediated killing, leading to an altered NK cell phenotype and function. For instance, changes in NK cell activation receptors or cellular exhaustion have been observed in melanoma patients, indicating that melanoma cells might exploit mechanisms to escape NK cell surveillance [8, 9].

The interaction between NK cells and the TME is complex and dynamic. Soluble factors such as TGF‐β, TNF‐α, PGE2, IL‐10, and IL‐12, as well as cell–cell interactions within the TME, can significantly influence NK cell functionality. These interactions can lead to phenotypic changes in NK cells, ranging from cytotoxic to exhausted or immunosuppressive states [10]. Recent studies have identified that NK cells infiltrating tumors exhibit distinct gene expression profiles, revealing specialized functional clusters with varying degrees of cytotoxicity and chemokine production [11].

Understanding how these NK cell characteristics correlate with patient outcomes is crucial for developing effective treatment strategies. This study aims to investigate whether melanoma patients can be classified into distinct subgroups with varying NK cell characteristics and to uncover their associations with treatment responses and prognostic outcomes. This approach could provide valuable insights into tailoring immunotherapy strategies and improving personalized treatment plans for skin melanoma patients.

## 2 Methods

### 2.1 Data Collection and Processing

A total of 244 NK‐cell‐related genes (NKRGs) were curated from previous literature sources [12, 13] and from gene sets associated with NK cell pathways in the Molecular Signatures Database (MSigDB) (http://www.gsea-msigdb.org/gsea/msigdb). The selection criteria for these genes included their documented involvement in NK cell differentiation, activation, and cytotoxic functions. Transcriptomic data for skin cutaneous melanoma (SKCM) patients were retrieved from The Cancer Genome Atlas (TCGA) via the Genomic Data Commons (GDC) portal (https://portal.gdc.cancer.gov/). These data were subsequently normalized using the DESeq 2 package, following standard procedures. Briefly, normalization included the estimation of size factors to account for sequencing depth and RNA composition, followed by variance stabilizing transformation to stabilize variance across genes and samples. Only genes with a minimum expression threshold (at least 10 raw counts in more than 50% of the samples) were retained for downstream analysis to reduce noise.

Single‐cell RNA sequencing data from cutaneous melanoma were obtained from the GSE215120 data set hosted on the Gene Expression Omnibus (GEO) platform (https://www.ncbi.nlm.nih.gov/geo/). The data were processed using the Seurat package (v4.3.0) in R. Preprocessing included quality control steps to filter out cells with fewer than 500 detected genes or with > 10% mitochondrial gene expression, to exclude low‐quality cells and doublets. After log‐normalization, the data set was scaled and the 2000 most variable genes were identified for downstream analysis. Principal component analysis was performed to reduce dimensionality, and the first 15 principal components were used for clustering using the Louvain algorithm (resolution parameter set to 0.8). Cluster markers were identified using the Wilcoxon rank‐sum test, and cell populations were annotated based on canonical marker expression.

To validate the primary findings, bulk RNA sequencing data from 44 cutaneous melanoma patients available in the GSE19234 data set, and 22 cutaneous melanoma patients available in the GSE65904 data set, were analyzed. Additionally, to assess the response to immunotherapy, transcriptomic data from 20 cutaneous melanoma patients who received immunotherapy, along with associated response data, were obtained from the GSE78220 data set. Another data set GSE244982 which included 31 cutaneous melanoma patients who demonstrated resistance to immunotherapy was also included to further validate the findings.

### 2.2 Unsupervised Clustering Analysis

Unsupervised clustering analysis was performed using the ConsensusClusterPlus package in R to identify molecular subtypes based on the expression profiles of NKRGs. Before clustering, the gene expression data were standardized by scaling each gene to have a mean of 0 and a standard deviation of 1, ensuring comparability across features. The clustering algorithm was run with the following parameters: the maximum number of clusters (maxK) was set to 8, the number of iterations (reps) was set to 1,000 to ensure stability, 80% of the samples (pItem) were randomly resampled during each iteration to account for noise, hierarchical clustering with complete linkage (clusterAlg = “hc”) was used, and Pearson correlation was employed as the distance metric (distance = “Pearson”). The optimal number of clusters was determined by evaluating the consensus matrix heatmaps for clear block structures, inspecting cumulative distribution function (CDF) curves for cluster stability, and calculating the area under the CDF curve to identify the point where stability gains plateaued. The robustness of the identified clusters was further assessed through silhouette analysis to evaluate compactness and separation, while cluster‐specific gene expression patterns were visualized using heatmaps to confirm distinct molecular characteristics.

### 2.3 GSVA Analysis

Gene Set Variation Analysis (GSVA) was performed using the GSVA package in R to assess the activity of predefined gene sets, with a particular focus on Gene Ontology pathways, across transcriptomic data for individual patients. The GSVA algorithm, a non‐parametric, unsupervised method, was applied to calculate pathway enrichment scores on a per‐sample basis, allowing continuous estimates of pathway activity rather than discrete assignments. The analysis was executed with the parameter kcdf = “Gaussian,” which assumes a continuous distribution of expression values suitable for RNA‐Seq data after normalization. GO pathways relevant to NK cell biology, including those involved in cytotoxicity, cytokine signaling, and immune regulation, were extracted based on prior curation and their relevance to the study's objectives. The differential activity of NK‐cell‐related pathways between patient subgroups was assessed. Visualization of the results was carried out using heatmaps and ridge plots to illustrate the variation in pathway activity across patient groups.

### 2.4 NK Cell Infiltration Analysis

NK cell infiltration levels were analyzed using the MCPcounter algorithm, a robust computational tool designed to estimate the abundance of various immune and stromal cell populations within the TME from transcriptomic data. The input for MCPcounter consisted of normalized expression data. The algorithm employs a curated set of marker genes specific to each cell type, including NK cells, to compute cell population scores that reflect their relative infiltration levels. The resulting NK cell infiltration scores were correlated with clinical outcomes, such as survival rates. Additionally, comparisons between patient subgroups were conducted using non‐parametric statistical tests to assess the significance of differences in NK cell infiltration levels.

### 2.5 Prediction of Immunotherapy Response

The normalized and standardized gene expression profiles were analyzed using the Tumor Immune Dysfunction and Exclusion (TIDE) framework (http://tide.dfci.harvard.edu/) to predict patients' responses to immune checkpoint blockade therapy [14]. The TIDE algorithm integrates two main components: the dysfunction score, which quantifies the functional impairment of tumor‐infiltrating T cells, and the exclusion score, which evaluates the extent of immune cell exclusion from the TME due to factors such as immunosuppressive cells, extracellular matrix components, or physical barriers. The input expression profiles were preprocessed to ensure compatibility with the TIDE platform by mapping gene identifiers to the required format and verifying data set integrity. TIDE scores were computed for each patient sample, and these scores were interpreted as composite indicators of immune evasion.

### 2.6 Assessment of NK Cell Exhaustion

NK cell exhaustion was evaluated by assessing the expression levels of specific exhaustion markers, including the activating receptor CD226, transcription factors EOMES and TBX21 (T‐bet), and cytotoxic molecules such as IFNG (interferon‐γ), TNF (tumor necrosis factor), PFN1 (perforin), and GZMA (granzyme A). A decrease in the expression of these markers is indicative of NK cell exhaustion, reflecting a reduced cytotoxic potential and impaired immune response within the TME.

### 2.7 Prediction of Drug Sensitivity

The sensitivity of each TCGA‐SKCM patient to various anticancer drugs was predicted using the oncoPredict package in R [15], which employs ridge regression models trained on the Genomics of Drug Sensitivity in Cancer (GDSC) database to estimate drug response profiles based on transcriptomic data. Before analysis, the RNA‐Seq data were normalized and log‐transformed to match the input requirements of the algorithm. Gene expression features from patient samples were aligned to the features used in the GDSC data set to ensure consistency. Ridge regression coefficients derived from the GDSC models were applied to the patient transcriptomic data to calculate estimated half‐maximal inhibitory concentration (IC50) values for each drug, where lower IC50 values indicate higher predicted sensitivity.

### 2.8 Development of a Gene Classifier for Cluster Discrimination

To develop a gene classifier that distinguishes between clusters, univariate Cox regression analysis was first conducted on NKRGs using the survival and survminer packages, selecting genes most significantly associated with prognosis (*p* < 0.001). These selected genes were then used as independent variables, with the cluster distribution as the dependent variable, in a Least Absolute Shrinkage and Selection Operator (LASSO) regression analysis. LASSO regression was performed using the glmnet package to address potential multicollinearity among predictors and to select the most predictive genes while applying a regularization penalty. The optimal lambda parameter, which controls the degree of shrinkage, was determined through 10‐fold cross‐validation to minimize prediction error. This process resulted in a final set of genes, each assigned a regression coefficient reflecting their contribution to the classifier. The classifier score was calculated by summing the expression levels of these genes, each weighted by their respective coefficients. Higher scores were indicative of a greater likelihood of belonging to Cluster 2, whereas lower scores suggested a propensity for Cluster 1. The predictive performance of the classifier was evaluated using receiver operating characteristic (ROC) curve analysis with the pROC package and calibration curves fitted with the rms package.

### 2.9 Statistical Analysis

Survival analysis was performed using the survival and survminer packages, with survival curves plotted using the survfit function and statistical significance assessed by the logrank test. Stacked bar plots were generated using the ggplot2 package, while heatmaps were created with the pheatmap package. Boxplots with overlaid scatter plots were produced using the ggpubr and ggplot2 packages, and ridge plots were generated using the ggridges package. Pearson correlation analysis was conducted using the cor. test function, with correlation scatter plots visualized using ggplot2. Sankey diagrams were drawn using the ggalluvial package. All data analyses were conducted in R version 4.3.0, with a *p*‐value < 0.05 considered statistically significant.

## 3 Results

### 3.1 Distinct NK‐Cell‐Related Clusters in SKCM

**Figure 1:**
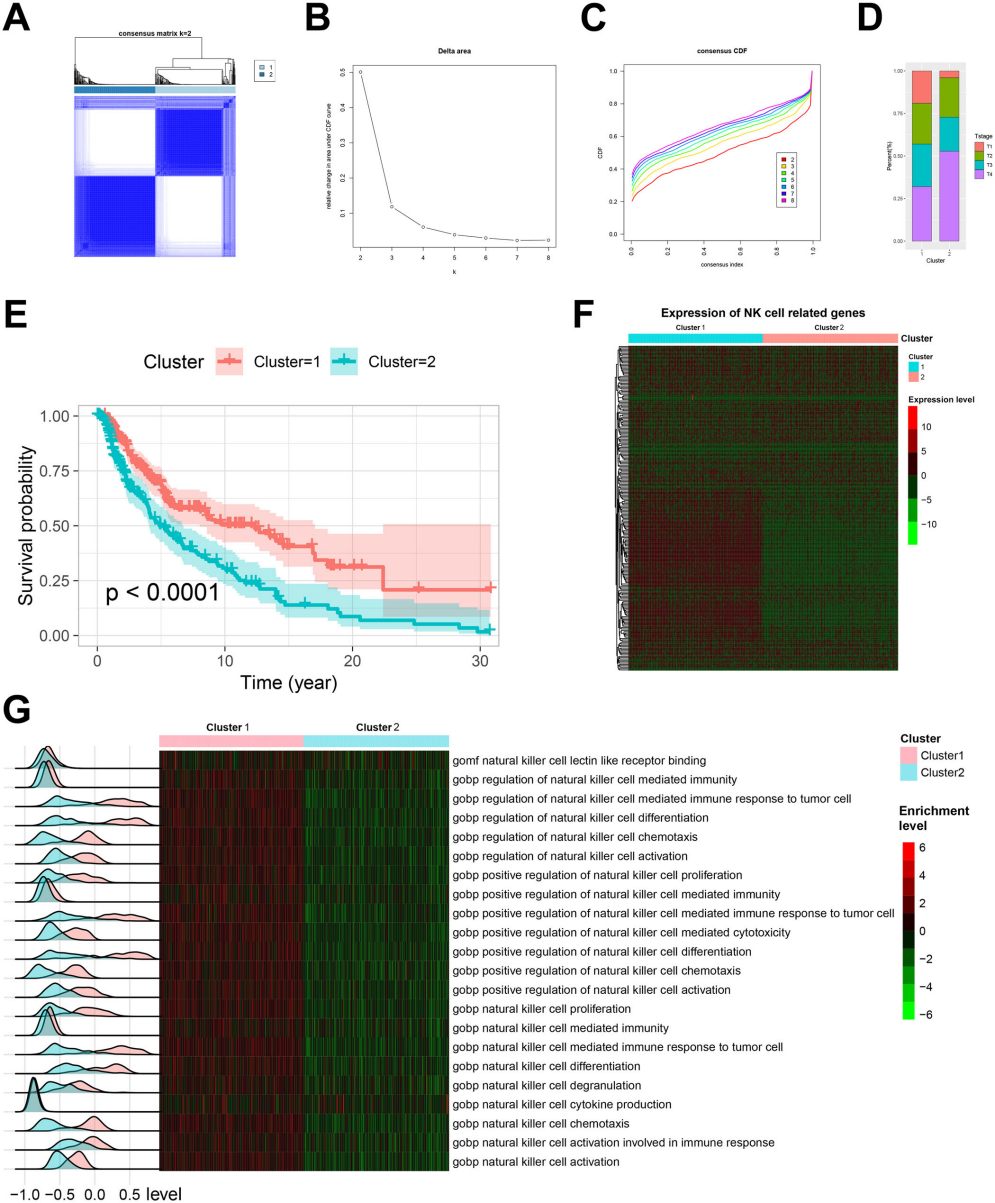
Distinct NK‐cell‐related clusters in SKCM and their clinical and molecular characteristics. (A) Consensus matrix for *k* = 2, showing the clear separation of TCGA‐SKCM patients into two distinct clusters based on natural killer‐cell‐related genes (NKRGs). The strong delineation between clusters reflects significant differences and a robust grouping of patients into specific subtypes. (B) Change in the area under the CDF curve as k increases. The sharp decline in the change beyond *k* = 2 indicates that *k* = 2 is the optimal number of clusters, as clustering stability decreases with higher *k* values. (C) Cumulative distribution function (CDF) curves for different k values, further supporting *k* = 2 as the optimal choice. The CDF plot demonstrates that, at *k* = 2, the clusters achieve maximal stability and differentiation. (D) Stacked bar plot comparing the distribution of tumor T stages between the two clusters. Cluster 1 has a larger proportion of patients at T1 stage and a smaller proportion at T4 stage, suggesting the potential clinical relevance of these clusters. (E) Kaplan–Meier survival curves showing overall survival (OS) differences between the two clusters. Patients in Cluster 1 (red) exhibit significantly better survival outcomes compared to those in Cluster 2 (blue), with a *p*‐value < 0.0001. (F) Heatmap of NKRG expression levels across the two clusters. Cluster 1 shows consistently higher expression of nearly half the NKRGs compared to Cluster 2, indicating the differential activity of NK‐cell‐related genes between these groups. (G) Heatmap and ridge plot illustrating the enrichment of NK‐cell‐related pathways, including chemotaxis, activation, proliferation, cytokine production, and immune responses to tumor cells. Cluster 1 patients exhibit significantly enhanced enrichment of these pathways (red scale) compared to Cluster 2. The ridge plots on the left also show that the peaks of pathway enrichment for Cluster 1 are consistently shifted to the right, reflecting stronger activation of NK cell functions in these patients.

Unsupervised clustering analysis based on NKRGs revealed that the majority of TCGA‐SKCM patients could be categorized into two distinct clusters (Figure 1A). The consensus matrix for *k* = 2 demonstrated clear separation between these clusters, with the change in area under the CDF curve (Figure 1B) and the CDF curve (Figure 1C) indicating that *k* = 2 was the optimal number of clusters. Notably, the distribution of tumor T stages differed significantly between the two clusters (Figure 1D); Cluster 2 had a higher proportion of T4 stage tumors and a lower proportion of T1 stage tumors compared to Cluster 1. Moreover, survival analysis revealed that patients in Cluster 1 had significantly better overall survival (OS) than those in Cluster 2 (*p* < 0.0001, Figure 1E). The expression patterns of NKRGs also varied markedly between the two clusters (Figure 1F). GSVA further highlighted that pathways associated with NK cell activity, including chemotaxis, activation, proliferation, cytokine production, and immune response to tumor cells, were significantly enriched in patients from Cluster 1 (Figure 1G). These findings suggest the existence of two distinct patient subgroups within SKCM, characterized by differential NKRG expression profiles and variations in NK cell activity.

### 3.2 Association of Clusters With NK Cell Levels and Immune Therapy Response

**Figure 2:**
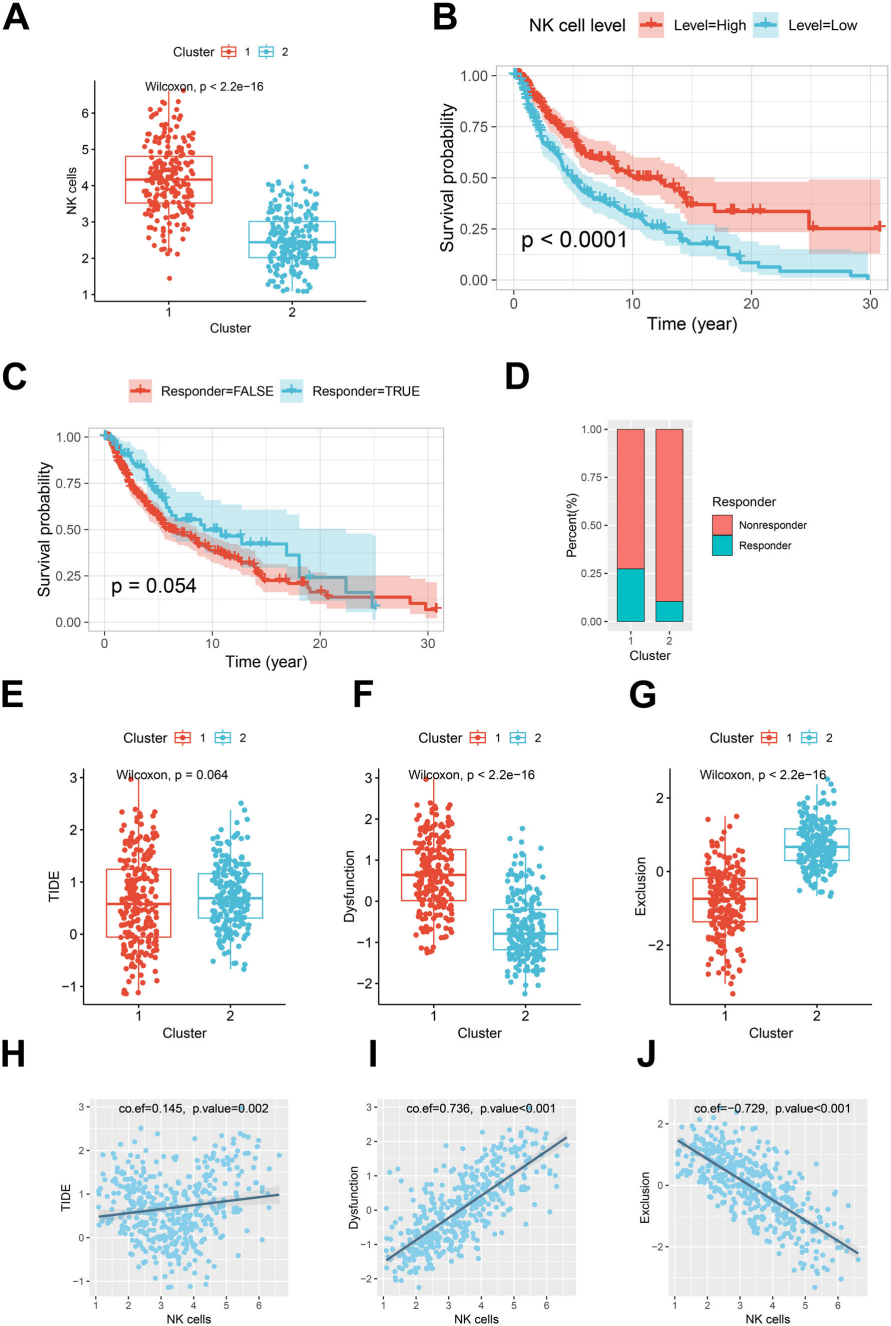
Associations between NK cell levels, immune therapy response, and cluster characteristics. (A) Boxplot of NK cell levels calculated by MCPcounter across the two clusters. Patients in Cluster 1 exhibit significantly higher NK cell levels compared to those in Cluster 2 (Wilcoxon, *p* < 2.2e − 16). (B) Kaplan–Meier survival curves showing overall survival (OS) stratified by NK cell levels. The median NK cell level in the TCGA‐SKCM cohort was used as the cutoff to classify patients into two groups. The red line represents patients with higher NK cell levels, who have better OS compared to those with lower NK cell levels (blue line, *p* < 0.0001). (C) Kaplan–Meier survival curves comparing predicted immune therapy responders (blue) and non‐responders (red). Predicted responders exhibit improved OS, though the difference does not reach statistical significance (*p* = 0.054). (D): Bar plot showing the proportion of predicted immune therapy responders in each cluster. Cluster 1 (left bar) has a significantly higher proportion of responders compared to Cluster 2 (right bar). (E) Boxplot of TIDE (Tumor Immune Dysfunction and Exclusion) scores for the two clusters. Cluster 1 has a marginally lower TIDE score compared to Cluster 2 (*p* = 0.064). (F) Boxplot of dysfunction scores for the two clusters. Patients in Cluster 2 exhibit significantly lower dysfunction scores than those in Cluster 1 (*p* < 2.2e − 16). (G) Boxplot of exclusion scores for the two clusters. Cluster 1 shows significantly lower exclusion scores compared to Cluster 1 (*p* < 2.2e − 16). (H) Scatter plot showing the positive correlation between NK cell levels (x‐axis) and TIDE scores (y‐axis). The correlation coefficient is 0.145 with *p* = 0.002. (I) Scatter plot illustrating the strong positive correlation between NK cell levels (x‐axis) and dysfunction scores (y‐axis). The correlation coefficient is 0.736 with *p* < 0.001. (J) Scatter plot showing the strong inverse correlation between NK cell levels (x‐axis) and exclusion scores (y‐axis). The correlation coefficient is −0.729 with *p* < 0.001.

Further analysis of NK cell infiltration levels revealed that NK cell levels, as calculated by the MCPcounter algorithm, were significantly higher in Cluster 1 compared to Cluster 2 (Figure 2A). Higher NK cell levels were associated with better OS, with patients exhibiting elevated NK cell levels showing significantly improved OS (*p* < 0.0001, Figure 2B).

TIDE analysis was employed to predict immune therapy responses for all TCGA‐SKCM patients. Predicted responders had better OS compared to non‐responders, approaching statistical significance (*p* = 0.054, Figure 2C). Additionally, Cluster 1 harbored a higher proportion of predicted responders compared to Cluster 2 (Figure 2D). There was no significant difference in TIDE scores between the clusters. However, Cluster 1 exhibited significantly higher dysfunction scores and lower exclusion scores compared to Cluster 2 (Figures 2E–2G). Correlation analysis demonstrated a weak but significant correlation between NK cell levels and the TIDE score (Figure 2H), a strong positive correlation with the dysfunction score (Figure 2I), and a strong negative correlation with the exclusion score (Figure 2J). These results suggest that the two NK cell‐related clusters differ markedly in NK cell expression levels and may have distinct associations with immune therapy response and prognosis.

### 3.3 Analysis of NK Cell Exhaustion Markers in the Two Clusters

**Figure 3:**
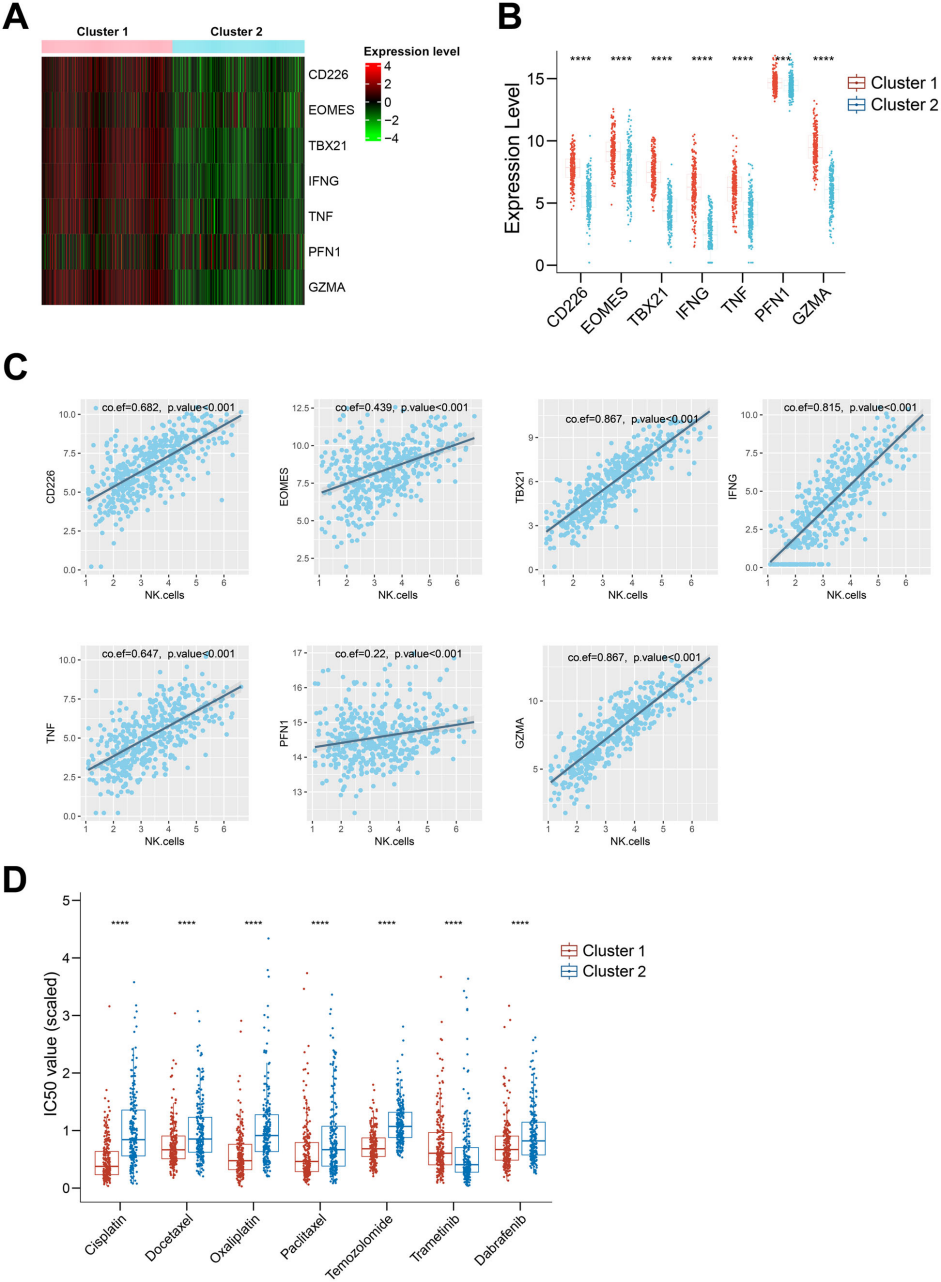
NK cell exhaustion markers and drug sensitivity of melanoma treatments in the two clusters. (A) Heatmap showing the expression levels of NK cell exhaustion markers in Cluster 1 and Cluster 2. Red lines indicate higher gene expression levels of these markers, while green lines indicate lower expression levels. (B) Boxplot illustrating significant differences in exhaustion marker levels between the two clusters. Each red dot represents the gene expression level of a patient in Cluster 1, and each blue dot represents a patient in Cluster 2. (C) Scatter plots demonstrating the positive correlations between NK cell levels and exhaustion marker levels. The x‐axis represents NK cell levels, and the y‐axis represents the marker genes. Each dot represents a patient. (D) Boxplot showing the IC50 values for various melanoma drugs in Cluster 1 and Cluster 2, highlighting differences in drug sensitivity. Each red dot represents the predicted IC50 value of a patient in Cluster 1 for the corresponding drug, and each blue dot represents a patient in Cluster 2.

Analysis of NK cell exhaustion markers revealed that all examined markers were expressed at higher levels in Cluster 1 compared to Cluster 2 (Figure 3A). Differences in these markers between the two clusters were statistically significant (*p* < 0.001 for all markers, Figure 3B). Furthermore, NK cell levels showed significant positive correlations with the levels of these exhaustion markers (Figure 3C; *p* < 0.001 for all correlations). These findings suggest that patients in the two clusters exhibit different degrees of NK cell exhaustion. Specifically, Cluster 2 not only has fewer NK cells and lower NK cell activity but also demonstrates more pronounced NK cell exhaustion.

### 3.4 Drug Sensitivity Analysis for Available Anticancer Agents of SKCM in the Two Clusters

Individual‐level predictive analysis of drug sensitivity in skin melanoma revealed that Cluster 1 exhibited significantly lower IC50 values for most of the available melanoma treatments, indicating higher drug sensitivity. This includes drugs such as cisplatin, docetaxel, oxaliplatin, paclitaxel, temozolomide, and dabrafenib (Figure 3D). Conversely, Cluster 2, which is associated with a poorer prognosis, showed significantly lower sensitivity to these drugs (*p* < 0.001). Notably, Cluster 2 displayed significantly lower IC50 values (better sensitivity) for trametinib compared to Cluster 1.

### 3.5 Distribution of NK Cell Exhaustion Markers in Single‐Cell Data

Single‐cell RNA sequencing data identified six distinct cell clusters (Figure S1). Expression of most NK cell exhaustion markers was predominantly observed within the NK cell cluster, though the distribution of these markers was heterogeneous among NK cells, with not all cells exhibiting similar expression levels. Notably, the PFN1 gene was uniformly expressed across all cell clusters.

### 3.6 Development of a Gene Classifier to Distinguish Between Clusters

**Figure 4:**
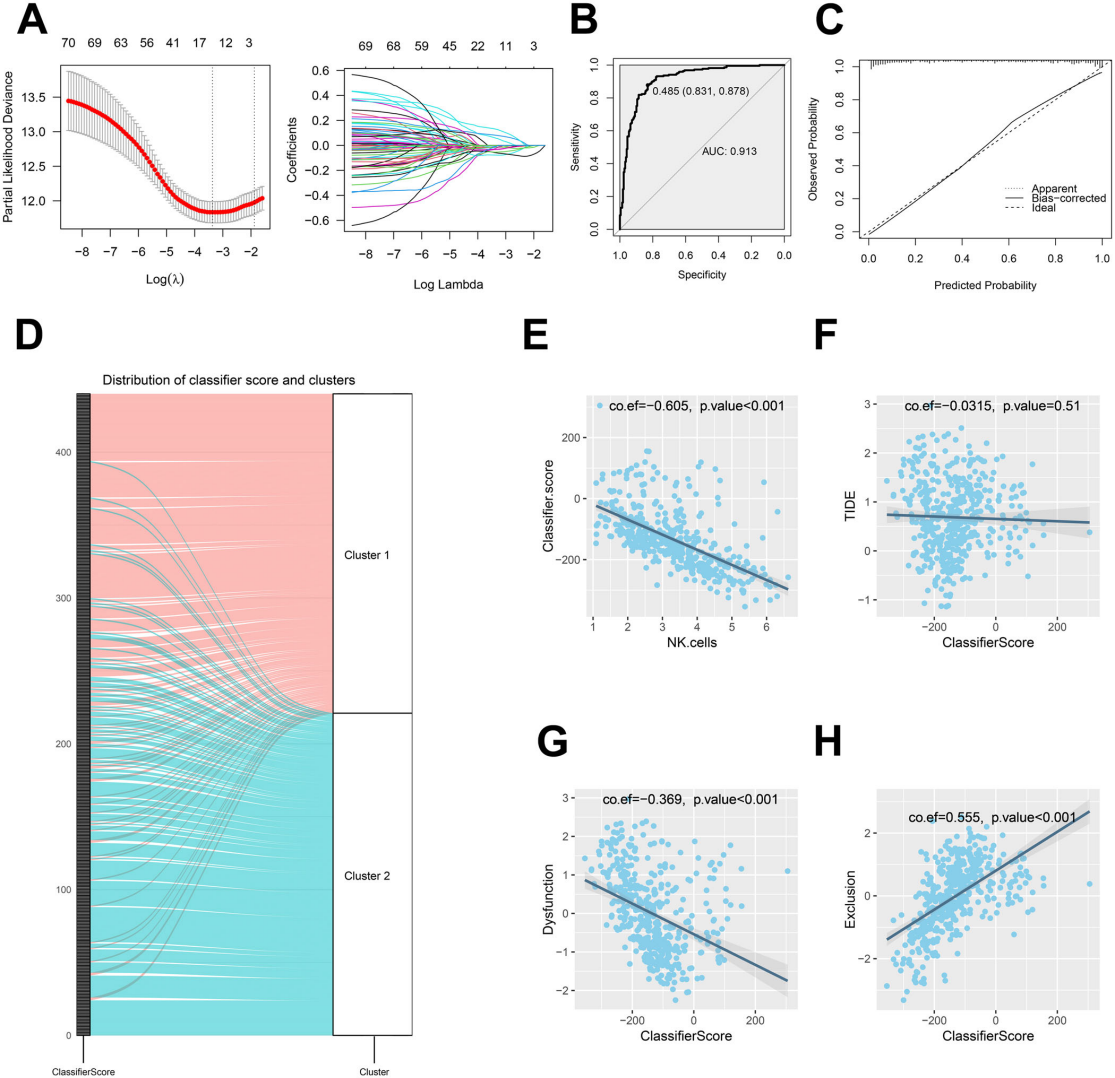
Development and validation of the gene classifier. (A) LASSO analysis for the gene classifier, showing deviance and coefficient plots. The x‐axis represents log(lambda), where larger values indicate greater penalization in the LASSO regression. The y‐axis on the left panel represents deviance, with lower values indicating a better‐fitting model. The y‐axis on the right panel represents coefficients. The top horizontal axis indicates the number of remaining variables as the penalization increases. The two vertical dashed lines represent the range of reasonable lambda values. (B) ROC curve demonstrating the predictive performance of the gene classifier with an AUC of 0.913. (C) Calibration curve assessing the classifier's calibration performance. The actual fitted curve closely follows the ideal result (diagonal line), indicating that the model predictions are well calibrated with reality. (D) Sankey diagram illustrating patient distribution into clusters based on classifier scores. The far‐left column represents all patients in the cohort, with scores gradually decreasing from top to bottom. The numbers on the left indicate the number of patients (from 100 to 200 to 300 to 400). Pink curves represent patients distributed in Cluster 1 (pink), and light blue curves represent patients distributed in Cluster 2 (light blue). This Sankey diagram clearly shows that most patients with higher scores (toward the top) are more likely to be distributed in Cluster 1 (pink). (E) Correlation scatter plot between classifier score and NK cell levels. Each dot represents a patient, with the x‐axis representing NK cell levels and the y‐axis representing the classifier score. The correlation coefficient is −0.605, and the *p*‐value is less than 0.001, indicating a significant negative correlation. (F) Correlation scatter plot between classifier score and TIDE score. Each dot represents a patient, with the x‐axis representing the classifier score and the y‐axis representing the TIDE score. The correlation coefficient is −0.0315, and the *p*‐value is 0.51, indicating no significant correlation. (G) Correlation scatter plot between classifier score and dysfunction score. Each dot represents a patient, with the x‐axis representing the classifier score and the y‐axis representing the dysfunction score. The correlation coefficient is −0.369, and the *p*‐value is less than 0.001, indicating a significant negative correlation. (H) Correlation scatter plot between classifier score and exclusion score. Each dot represents a patient, with the x‐axis representing the classifier score and the y‐axis representing the exclusion score. The correlation coefficient is 0.555, and the *p*‐value is less than 0.001, indicating a significant positive correlation.

A total of 71 genes significantly associated with OS were selected through univariate analysis. Subsequently, a gene classifier was established using LASSO analysis (Figure 4A), with the classifier formula defined as: ClassifierScore = 6.0806 × KLRC1 + (−1.2094) × KLRC2 + (−0.6148) × KLRD1 + (−2.5236) × IFNAR2 + (−0.3820) × IL15 + 1.7457 × PGLYRP3 + (−1.2174) × IL18RAP + 1.7984 × TUBB4B. The classifier score was computed for all TCGA‐SKCM patients to predict their cluster assignment. The ROC curve (Figure 4B) demonstrated high predictive performance of the classifier, with an AUC of 0.913. The calibration curve (Figure 4C) also indicated excellent calibration performance. The Sankey diagram (Figure 4D) illustrates that as the classifier score increases, patients are increasingly likely to be classified into Cluster 1, with only a few patients being misclassified.

Additionally, the classifier score showed a significant negative correlation with NK cell levels (Figure 4E). Although there was no significant correlation with the TIDE score (Figure 4F), it exhibited a significant negative correlation with the dysfunction score (Figure 4G) and a significant positive correlation with the exclusion score (Figure 4H).

### 3.7 External Validation of the Classifier in an Independent Data Set

**Figure 5:**
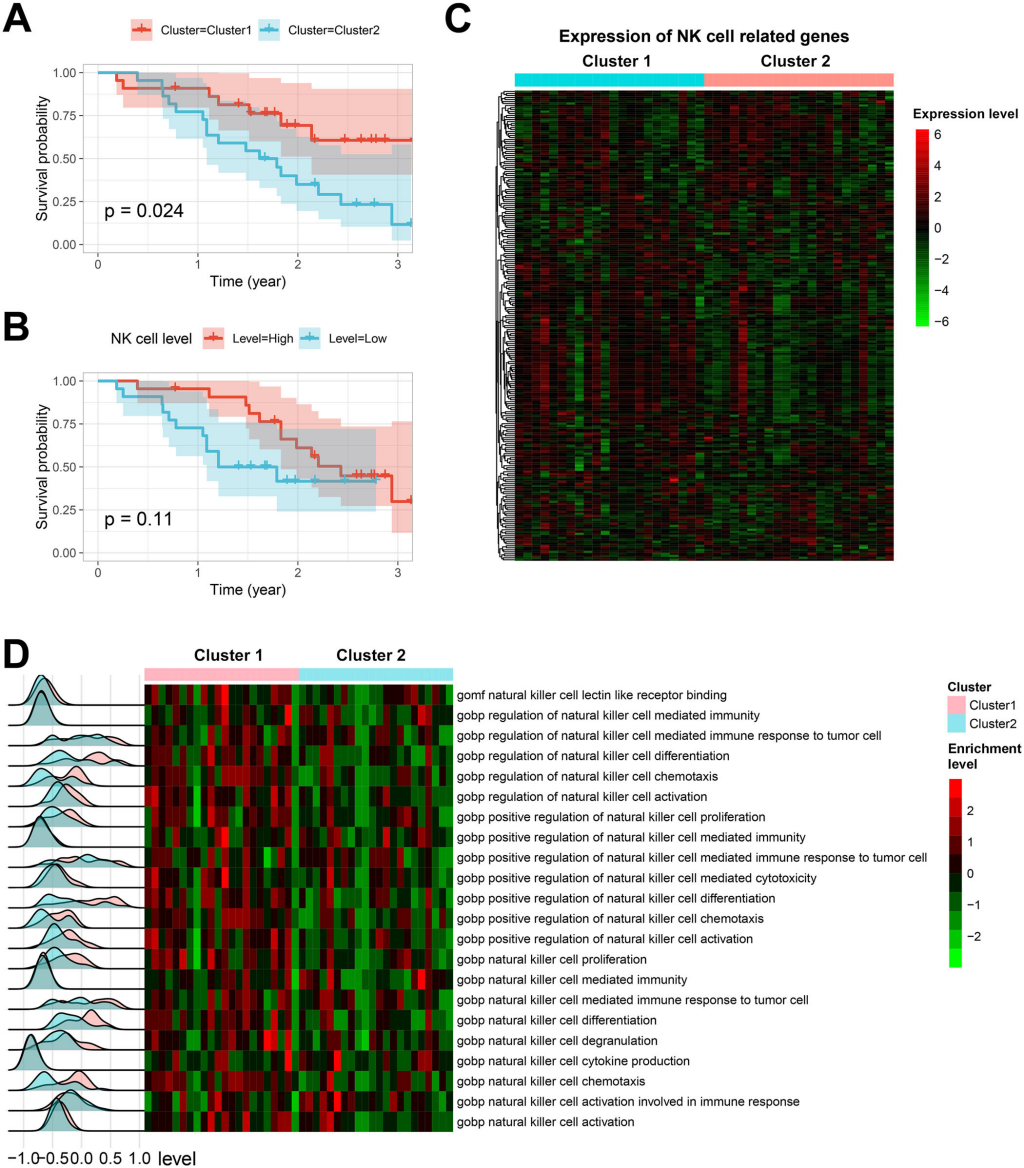
External validation of the gene classifier in the GSE19234 data set. (A) Survival curves comparing Cluster 1 and Cluster 2 based on classifier scores. The Kaplan–Meier survival curves show the survival probability over time for patients in Cluster 1 (red) and Cluster 2 (blue). The *p*‐value of 0.024 indicates a statistically significant difference in survival between the two clusters. (B) Survival curves for high and low NK cell levels. The Kaplan–Meier survival curves show the survival probability over time for patients with high NK cell levels (red) and low NK cell levels (blue). The *p*‐value of 0.11 indicates that there is no statistically significant difference in survival between the two groups. (C) Heatmap of NKRG expression profiles for Clusters 1 and 2. The heatmap shows the expression levels of NK‐cell‐related genes in Cluster 1 (cyan) and Cluster 2 (pink). Red indicates higher gene expression levels, while green indicates lower gene expression levels. (D) Ridge plot and heatmap from GSVA analysis of NK‐cell‐related pathways. The ridge plot on the left shows the distribution of pathway scores for different NK‐cell‐related pathways. The heatmap on the right shows the expression levels of these pathways in Cluster 1 (pink) and Cluster 2 (light blue). Each row represents a different pathway, and each column represents a patient. Red indicates higher pathway activity, while green indicates lower pathway activity.

In the external data set GSE19234, the classifier score was computed for each SKCM patient based on tumor expression profiles. Patients with scores above the median were classified into Cluster 1, while those with lower scores were assigned to Cluster 2. Survival analysis (Figure 5A) confirmed the initial findings, showing that Cluster 1 had a significantly better survival prognosis compared to Cluster 2 (*p* = 0.024).

NK cell infiltration levels in tumors from the GSE19234 data set were assessed using the MCPcounter algorithm. Survival curves (Figure 5B) for patients with high and low NK cell levels indicated that higher NK cell levels were associated with better prognosis, though the result approached statistical significance (*p *= 0.11). The NKRG expression profiles for the clusters identified by the classifier (Figure 5C) again demonstrated distinct NKRG expression characteristics between Cluster 1 and Cluster 2. GSVA analysis of NK cell‐related pathways, represented in ridge plots and heatmaps (Figure 5D), showed that Cluster 1 had higher enrichment in most NK cell activity pathways compared to Cluster 2.

The heatmap (Figure S2A) and boxplot (Figure S2B) of NK cell exhaustion markers indicated a greater degree of NK cell exhaustion in Cluster 2, although some markers lacked statistical significance due to smaller sample sizes. The correlation between NK cell levels and these markers remained consistent (Figure S2C). Consistent with previous findings, Cluster 1 in the GSE19234 data set was associated with higher dysfunction scores and lower exclusion scores (Figure S3A). Correlation scatter plots further supported these results (Figure S3B). These external validation results confirm that the gene classifier effectively identifies Cluster 1 and Cluster 2 across different patient populations, reinforcing the robustness of these findings.

These findings were further validated in another data set GSE65904. Cluster 1 as identified by the gene classifier showed a trend for improved prognosis, including distant metastasis‐free survival (DMFS) and disease‐specific survival (DSS) (Figure S4A). Cluster in GSE65904 also showed relatively higher expression in NKRGs (Figure S4B). Most of the NK cell activity pathways were more enriched in Cluster 1 (Figure S4C).

### 3.8 Validation of Classifier Scores for Immune Response Prediction

**Figure 6:**
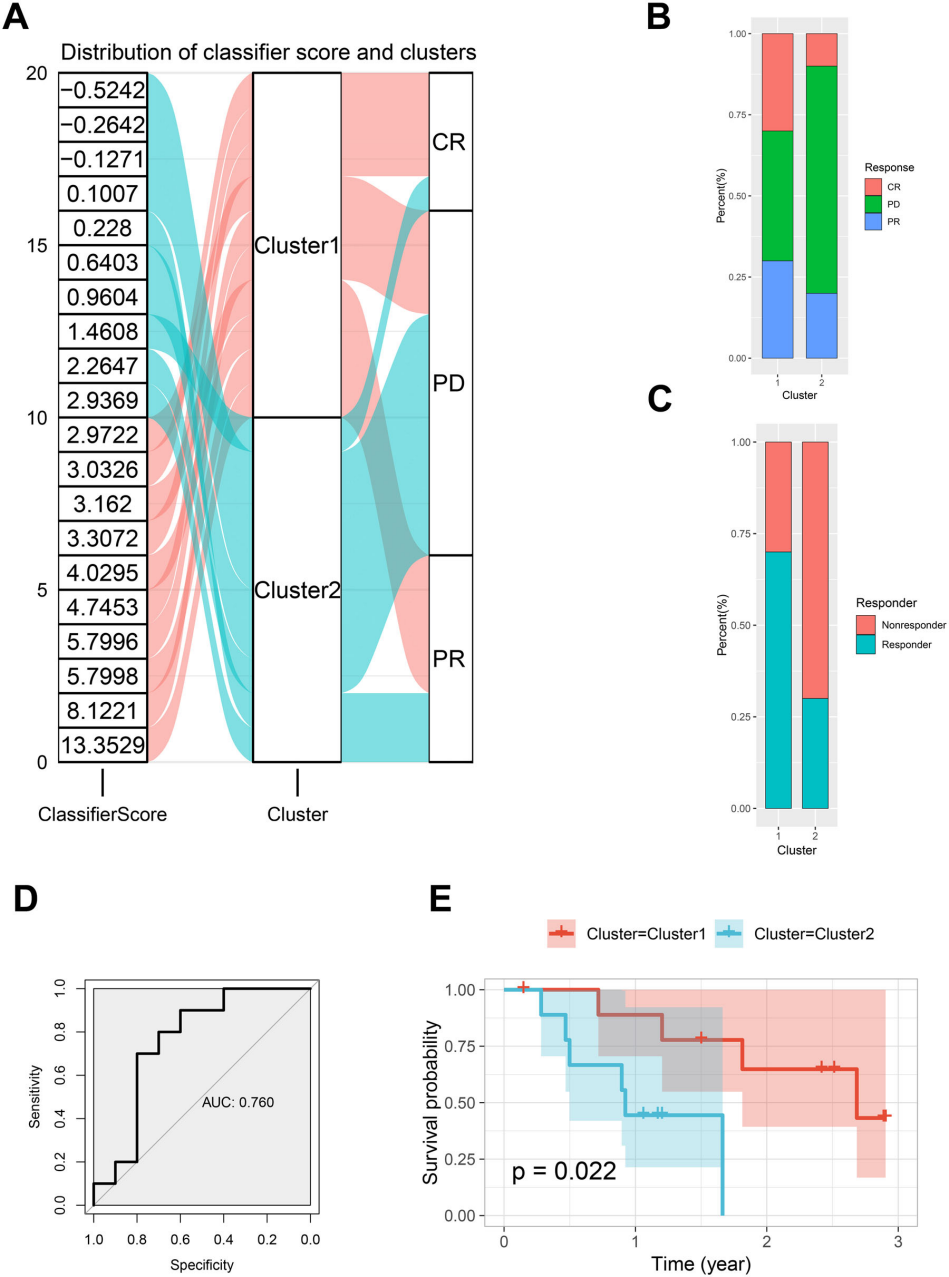
External validation of classifier scores in GSE78220 for Immunotherapy response. (A) Sankey diagram illustrating the distribution of immune response outcomes (CR, PD, PR) among patients in Cluster 1 and Cluster 2. The far‐left column represents all patients in the cohort, with classifier scores gradually increasing from top to bottom. The numbers on the left indicate the numbers of patients. Pink curves represent patients distributed in Cluster 1 (pink), and light blue curves represent patients distributed in Cluster 2 (light blue). This Sankey diagram clearly shows the distribution of complete response (CR), partial response (PR), and progressive disease (PD) outcomes across the two clusters. (B) Bar plot showing the rates of CR, PR, and PD for patients in Cluster 1 versus Cluster 2. Each bar is divided into three segments representing the percentage of patients with CR (red), PR (green), and PD (blue). The plot indicates the proportion of each response type in both clusters. (C) Bar plot depicting the responder rates (CR + PR) for Cluster 1 and Cluster 2, indicating higher responder rates in Cluster 1. Each bar is divided into two segments representing the percentage of responders (CR + PR) and non‐responders (PD). The plot highlights the higher responder rate in Cluster 1 compared to Cluster 2. (D) ROC curve assessing the classifier's ability to predict responder status, with an AUC of 0.76. (E) Survival curves showing overall survival (OS) for patients in Cluster 1 and Cluster 2, with Cluster 1 demonstrating significantly better survival outcomes (*p* = 0.022). The Kaplan–Meier survival curves show the survival probability over time for patients in Cluster 1 (red) and Cluster 2 (blue). The *p*‐value of 0.022 indicates a statistically significant difference in survival between the two clusters.

In an external data set, GSE78220, which includes data on SKCM patients' responses to immunotherapy, we applied the classifier scores to identify patients categorized into Cluster 1 and Cluster 2. Analysis of immune response outcomes (complete response (CR), progressive disease (PD), and partial response (PR)) is illustrated in Figure 6A. Patients in Cluster 1 exhibited a higher rate of CR and PR, and a lower rate of PD compared to those in Cluster 2. The bar plot in Figure 6B further shows that Cluster 1 has a significantly higher rate of CR and PR.

When considering both CR and PR as responders, Figure 6C indicates that Cluster 1 also has a greater proportion of responders compared to Cluster 2. The classifier's predictive capability for responders was evaluated with an area under the ROC curve of 0.76 (Figure 6D), demonstrating a strong association between the classifier‐defined clusters and immunotherapy response. Additionally, survival analysis (Figure 6E) confirms that the OS of Cluster 1 patients is significantly better than that of Cluster 2 (*p* = 0.022), supporting the predictive value of the classifier for immunotherapy response and prognosis.

To further validate the findings, the data set GSE244982 with 31 immunotherapy‐resistant patients was combined with the responders in GSE78220. The clusters as identified by the gene classifier demonstrated a consistent pattern to immunotherapy sensitivity, with Cluster 1 containing the majority of responders (Figure S5).

## 4 Discussion

This study offers a comprehensive analysis of the distinct biological and clinical features associated with two NK‐cell‐related clusters in skin cutaneous melanoma. Through an integrative approach combining unsupervised clustering, survival analysis, and external validation, we identified and characterized two distinct patient subgroups based on their NK cell gene expression profiles. Our findings reveal that Cluster 1 and Cluster 2 exhibit significant differences in NK cell activity, immune response, and clinical outcomes. Specifically, patients in Cluster 1 demonstrate higher NK cell infiltration and activity, more favorable overall survival, and better responses to immunotherapy compared to those in Cluster 2. These clusters also differ markedly in their expression of NK cell exhaustion markers and drug sensitivity profiles. This research also introduces a novel gene classifier that effectively discriminates between the two clusters, with implications for predicting patient prognosis and tailoring treatment strategies. By demonstrating that Cluster 1 patients are more likely to respond positively to immunotherapy, this study highlights the potential of NKRG signatures as biomarkers for personalized treatment approaches in melanoma. The findings underscore the importance of integrating molecular profiling into clinical practice to enhance patient stratification and optimize therapeutic outcomes.

Our analysis highlights the pivotal role of NK cells in melanoma. NK cells are essential components of the innate immune system, with a well‐documented ability to recognize and eliminate tumor cells. The higher NK cell infiltration observed in Cluster 1 correlates with improved overall survival (OS), supporting the hypothesis that a robust NK cell response is associated with a favorable prognosis. This finding aligns with existing literature that underscores the protective role of NK cells in melanoma by directly targeting tumor cells and enhancing adaptive immune responses [5, 6]. Moreover, our results indicate that patients in Cluster 1, with higher NK cell levels, exhibit better responses to immunotherapy compared to those in Cluster 2. This is particularly relevant given that immunotherapy has become a cornerstone in melanoma treatment. The enhanced response in Cluster 1 suggests that NK cell activity could be a key determinant of therapeutic efficacy. Previous studies have shown that NK cells interact with dendritic cells and secrete cytokines that help shape adaptive immune responses, thus potentially improving responses to therapies such as checkpoint inhibitors [5]. Conversely, Cluster 2 shows higher levels of NK cell exhaustion markers, which correlates with poorer outcomes. This suggests that melanoma's microenvironment may induce functional exhaustion of NK cells, impairing their ability to control tumor growth. NK cell exhaustion is a well‐documented phenomenon where persistent exposure to tumor antigens and immunosuppressive factors leads to diminished NK cell functionality [8, 10]. This study's findings reinforce the concept that melanoma cells can evade immune surveillance through mechanisms that include inducing NK cell exhaustion and altering the TME, making NK cells less effective at eliminating tumor cells [16].

Our data reveal that NK cell depletion markers are expressed variably across different NK cell clusters, suggesting that NK cell function and activation are heterogeneous within the TME. The differential expression of NK cell exhaustion markers and their correlation with clinical outcomes highlight the complexity of NK cell responses in melanoma. Specifically, our results show that Cluster 2, which is associated with a poorer prognosis, exhibits a higher level of NK cell exhaustion markers. This aligns with previous studies indicating that NK cell exhaustion and dysfunction are common in melanoma, likely due to chronic exposure to immunosuppressive factors within the TME [17, 18]. The interaction between NK cells and melanoma cells is influenced by several TME factors. Our findings that NK cell exhaustion markers are more prominent in the less favorable cluster underscore the impact of TME conditions on NK cell function. Melanoma cells can exploit TME components to inhibit NK cell activity, such as by upregulating inhibitory receptors or downregulating activating ones. This is consistent with the observation that TME‐associated factors like TGF‐β and PGE2 can suppress NK cell activity, reducing their efficacy in tumor elimination [18, 19]. Furthermore, our study highlights that NK‐cell‐related pathways are significantly altered between the two identified clusters. The higher NK cell activity in Cluster 1, associated with a better prognosis, contrasts with the lower NK cell activity in Cluster 2. This differential activity could be attributed to variations in the TME composition, such as the presence of immunosuppressive cytokines and other cells that interact with NK cells. For instance, CAFs and other TME components can produce factors that inhibit NK cell activation and cytotoxicity, reinforcing the immune escape mechanisms employed by melanoma cells [19, 20]. Our external validation in the GSE78220 data set, which demonstrates a higher responder rate to immunotherapy in Cluster 1, further supports the notion that NK cell activity correlates with treatment response. The fact that Cluster 1 patients exhibit a higher rate of complete and partial responses suggests that effective NK cell function is a critical determinant of favorable outcomes in melanoma treatment. This is in line with evidence suggesting that NK cells contribute significantly to the efficacy of immune therapies [21, 22]. The findings reveal how NK cells can be functionally impaired by TME factors and highlight the importance of considering NK cell status when evaluating melanoma prognosis and treatment responses. Enhancing NK cell activity or counteracting the suppressive elements of the TME could provide new avenues for improving therapeutic outcomes in melanoma.

Our study highlights the crucial role of NK cells in melanoma and identifies significant avenues for therapeutic intervention. We observed distinct differences in NK cell function and exhaustion between melanoma clusters, which suggests that targeting NK cell dysfunction could be a key strategy in enhancing treatment outcomes. NK cells are vital in the initial immune response against melanoma, yet their effectiveness is often compromised by the TME. Our findings, which show lower NK cell activity and increased exhaustion markers in Cluster 2, underscore the need to address these dysfunctions to improve therapeutic efficacy. Restoring NK cell function through cytokine therapy represents a promising approach. Specifically, IL‐15, which was noted to mitigate NK cell exhaustion in previous study, could enhance NK cell cytotoxicity and restore their antitumor activity [7]. In addition, engineering NK cells to express chimeric antigen receptors (CARs) targeting melanoma‐specific antigens could significantly enhance their efficacy. Our results support this by demonstrating that NK cells in Cluster 1, which exhibit more robust activation profiles, might benefit from such targeted approaches [18]. Moreover, therapeutic strategies aimed at modulating NK cell inhibitory receptors could further enhance their function. For instance, targeting NKG2A with monoclonal antibodies like monalizumab could reverse NK cell inhibition, as suggested by our data showing differential expression of NK cell receptors between clusters [18]. Furthermore, addressing soluble immune checkpoints, such as sMIC, which was found to correlate with poor responses to PD‐1/PD‐L1 blockade in melanoma patients, could improve treatment efficacy [23]. Overall, by combining cytokine therapy, CAR‐engineered NK cells, and immune checkpoint modulation, our study suggests several promising strategies to harness NK cells more effectively against melanoma. Notably, while Cluster 2 patients face significant challenges due to NK cell dysfunction and poor prognosis, their heightened sensitivity to trametinib offers a valuable treatment opportunity. This suggests that a tailored therapeutic approach, combining targeted therapies like trametinib with strategies to enhance NK cell activity, could be effective in improving outcomes for these patients.

This study has several limitations that should be acknowledged. First, while our study benefits from extensive data obtained from large public databases like TCGA and GEO, these resources may not fully reflect the wide diversity of melanoma patients across different ethnic backgrounds and geographic locations. The representation of Asian populations, particularly Chinese patients, is limited in these databases. This does not significantly impact the overall reliability of our findings but points to an area where further research could be beneficial. Second, the interactions within the TME are complex, and while we focused on NK cell markers and transcriptomic characteristics, other factors such as influences from different immune cells or changes in the local tumor environment also play significant roles. Our study highlights key aspects of NK cell behavior, yet acknowledges that additional elements can affect NK cell function. Lastly, our gene classifier for distinguishing patient subgroups and linking them to responses to immunotherapy and drug sensitivities relies on previously established data. The rapid emergence of novel antitumor drugs in recent years underscores the need for more up‐to‐date data to verify the accuracy of our predictions regarding drug sensitivity.

## 5 Conclusion

This study highlights the crucial role of NK cell profiles in melanoma, identifying two distinct clusters with differing prognoses and treatment responses. Cluster 1, with high NK cell activity, shows better response to immunotherapy and better survival outcomes, while Cluster 2, despite a poorer prognosis, is more responsive to trametinib. These findings suggest that NK cell‐based classification can inform personalized treatment strategies, offering potential for improved outcomes through targeted therapies tailored to individual patient profiles.

## Author Contributions

Conception and design: Jun Zhou, Renhui Cai, and Caifeng Chen. Collection and assembly of data: Jun Zhou, Renhui Cai, and Danqun Zhang. Data analysis and interpretation: Jun Zhou, Renhui Cai, and Danqun Zhang. Checking and confirmation of the authenticity of the raw data: Jun Zhou and Caifeng Chen. Manuscript writing: All authors. All authors have read and approved the final version of the manuscript.

## Ethics Statement

The authors have nothing to report.

## Consent

The authors have nothing to report.

## Conflicts of Interest

The authors declare no conflicts of interest.

## Patient and Public Involvement Statement

The authors have nothing to report.

## Supporting information

Supporting information.

Supporting information.

Supporting information.

Supporting information.

Supporting information.

Supporting information.

## Data Availability

All data generated or analyzed during this study are included in this article and its Supplementary material files. Further enquiries can be directed to the corresponding author.
