# Supplementary figures and images for: Identification of Natural Killer Cell‐Associated Clusters in Skin Melanoma and the Impact on Prognosis and Drug Sensitivity
Source: Immun Inflamm Dis. 2025 Feb 17;13(2):e70143. doi: 10.1002/iid3.70143 (PMC11831448; doi:10.1002/iid3.70143)

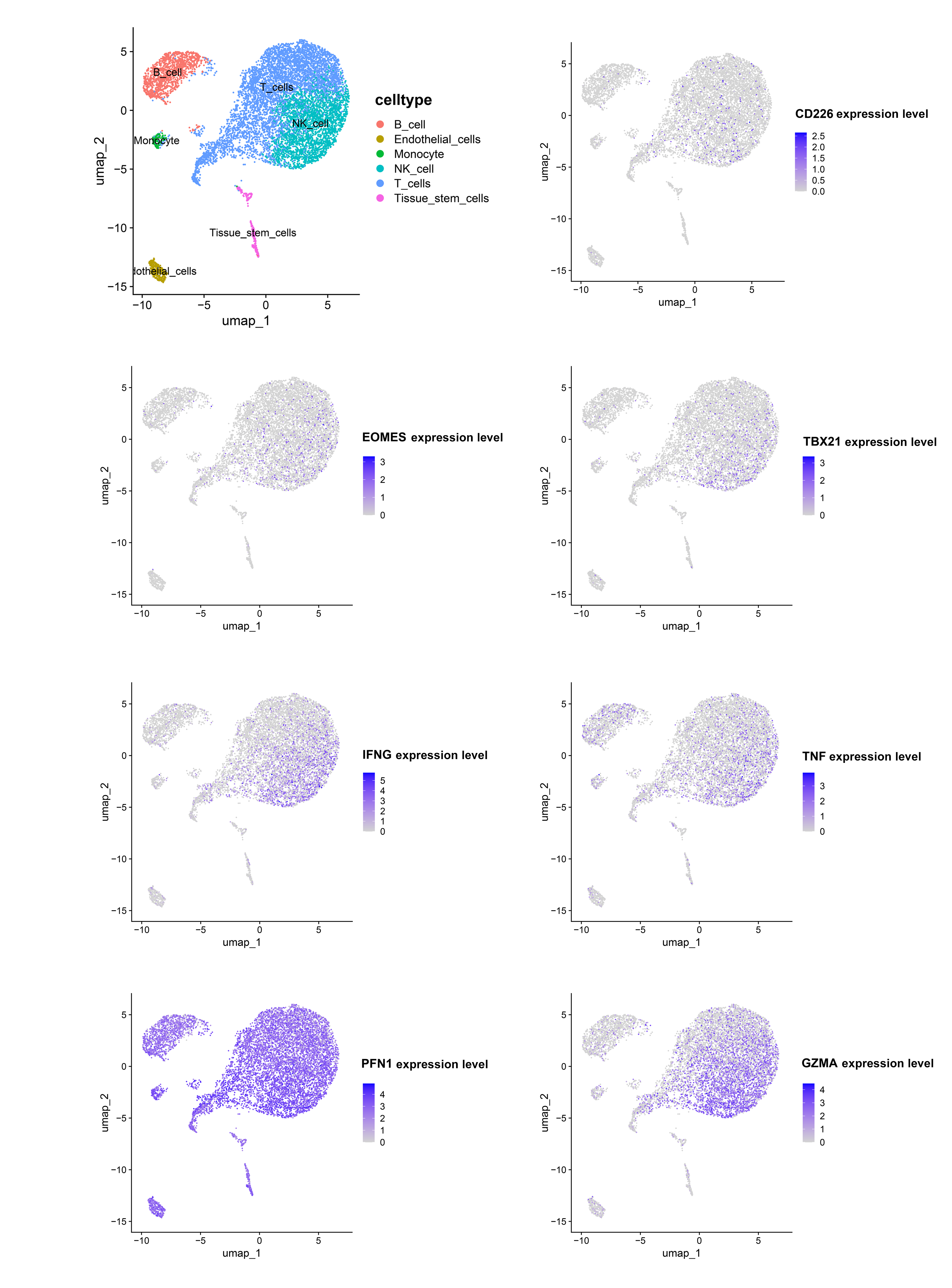

Supplement: Supplementary file 1 — Supporting information. [file IID3-13-e70143-s004.tif]

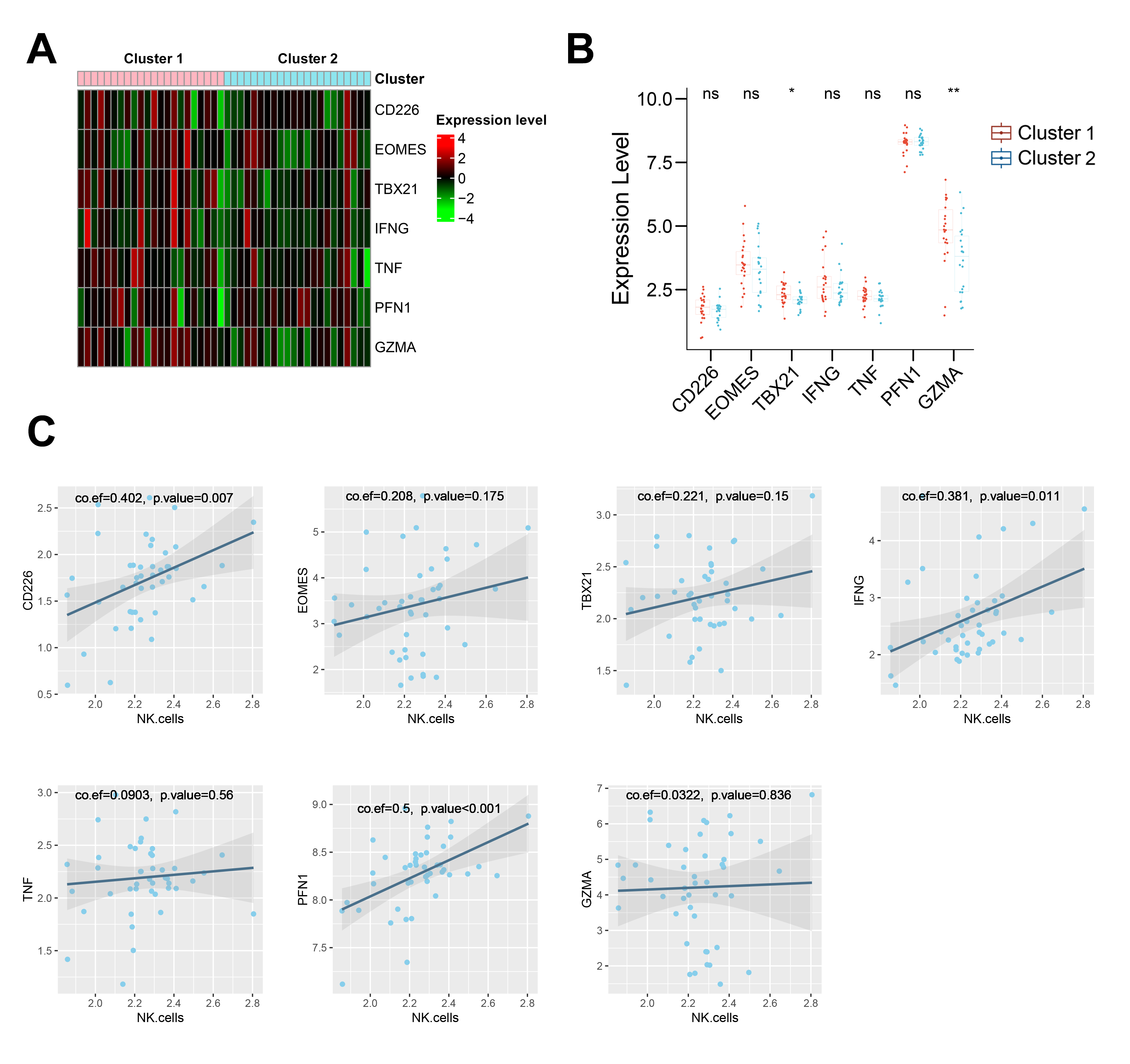

Supplement: Supplementary file 2 — Supporting information. [file IID3-13-e70143-s001.tif]

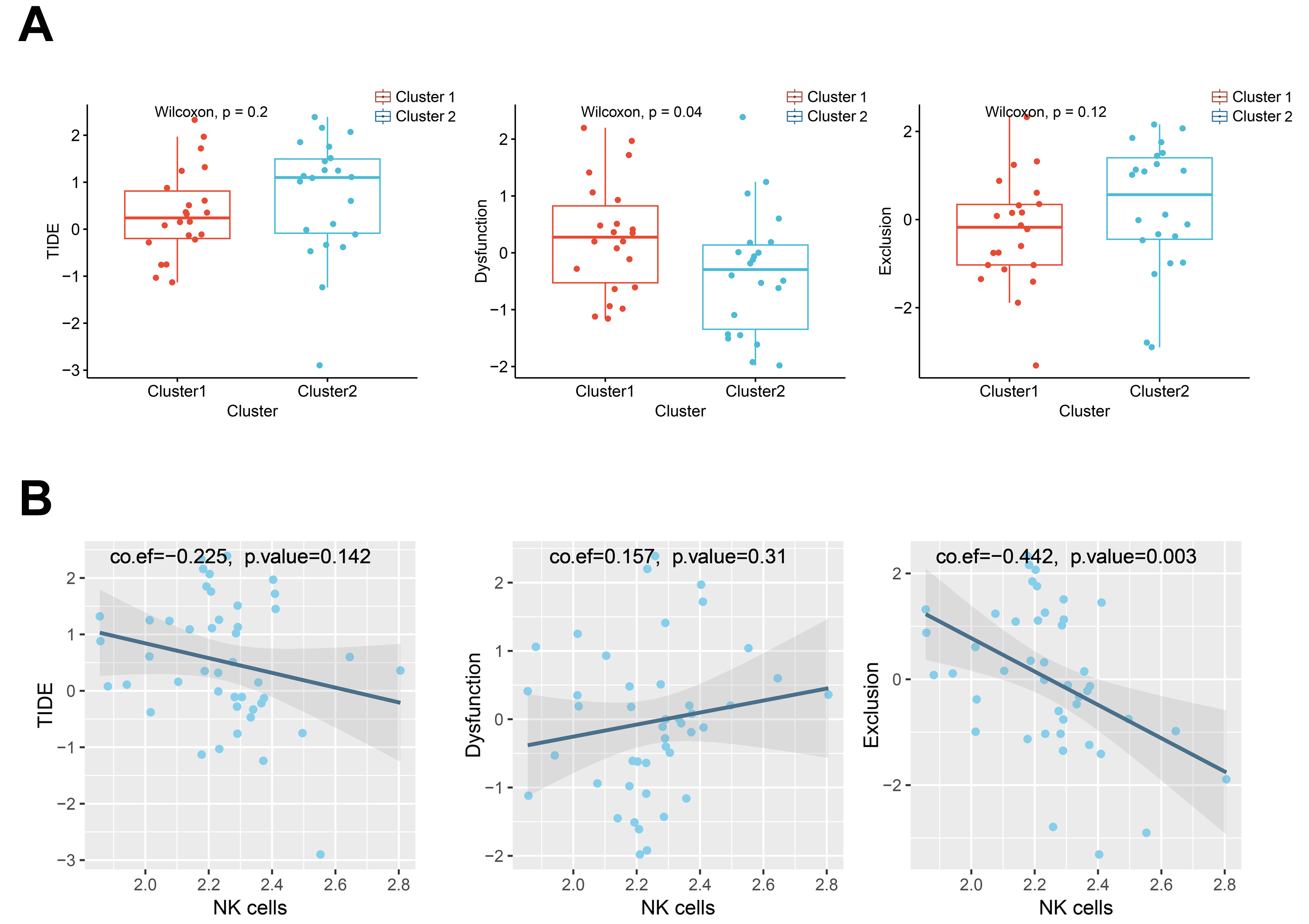

Supplement: Supplementary file 3 — Supporting information. [file IID3-13-e70143-s005.tif]

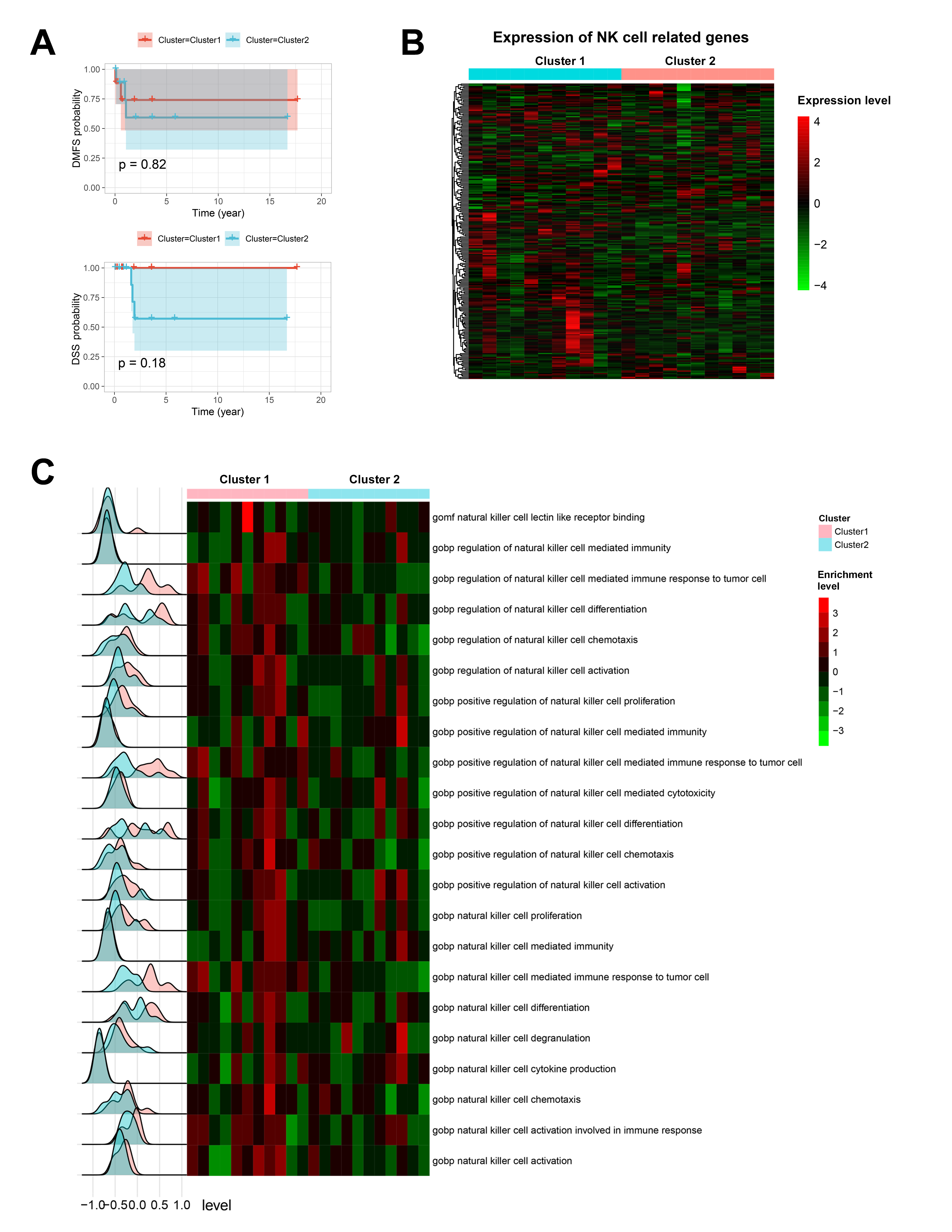

Supplement: Supplementary file 4 — Supporting information. [file IID3-13-e70143-s006.tif]

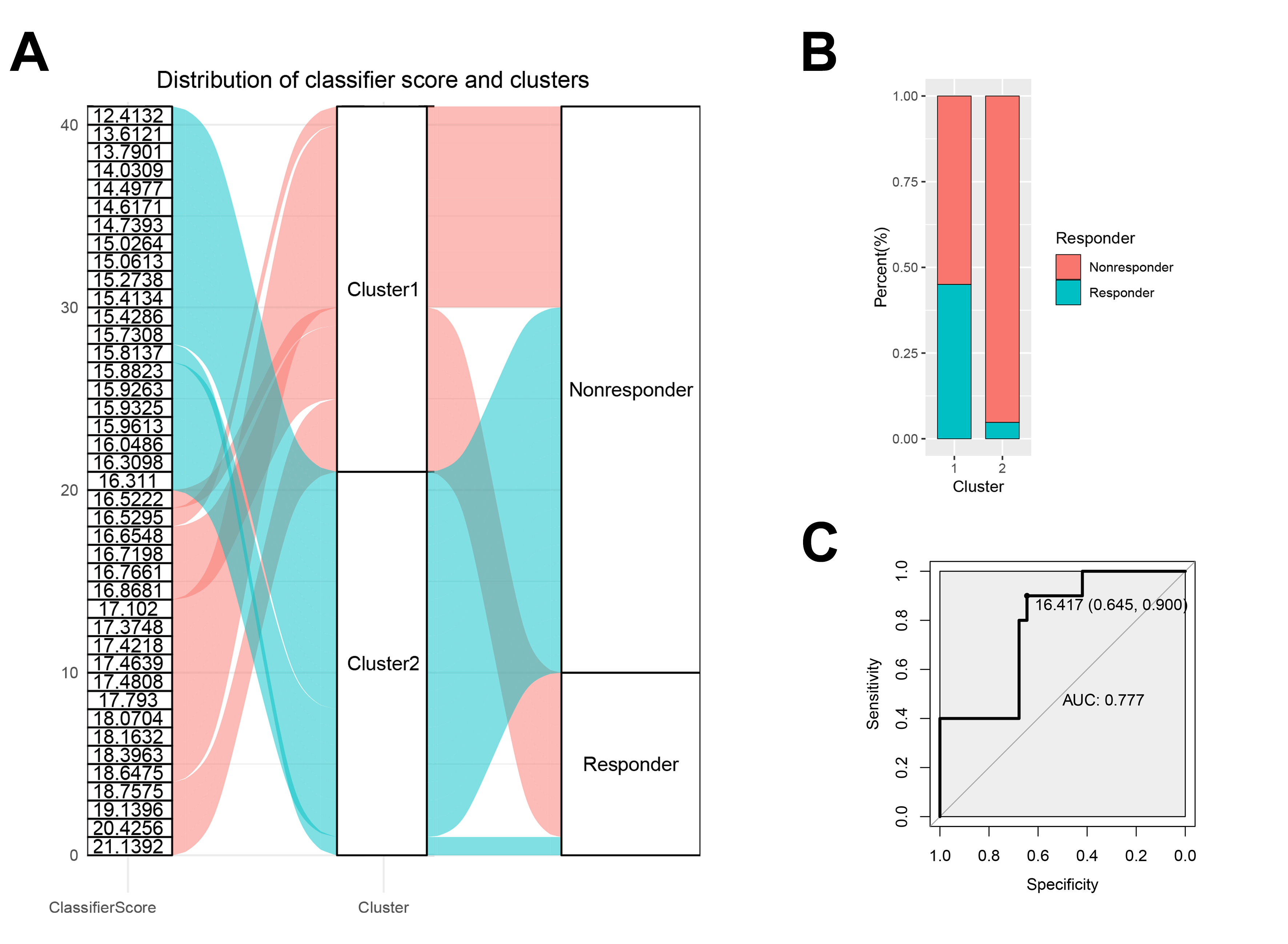

Supplement: Supplementary file 5 — Supporting information. [file IID3-13-e70143-s003.tif]
