# Supplementary material for: Identification of Natural Killer Cell‐Associated Clusters in Skin Melanoma and the Impact on Prognosis and Drug Sensitivity
Source: Immun Inflamm Dis. 2025 Feb 17;13(2):e70143. doi: 10.1002/iid3.70143 (PMC11831448; doi:10.1002/iid3.70143)
Supplement: Supplementary file 6 — Supporting information. [file IID3-13-e70143-s002.docx]

**Supplementary Figure S1. Distribution of NK Cell Exhaustion Markers in Single-Cell RNA Sequencing Data**

The first panel (left upper corner) shows the distribution of different cell types on a UMAP plot. Each cell type is represented by a different color. This UMAP plot serves as a reference for the subsequent gene expression distributions. The remaining panels show the expression patterns of NK cell exhaustion markers across these cell clusters. The expression levels are indicated by the color gradient, with darker blue representing higher expression levels. By comparing the gene expression distributions with the cell type distribution in the first panel, the cell types with higher expression levels for each marker gene can be identified.

**Supplementary Figure S2. External Validation of the NK Cell Exhaustion Markers in Gene Classifier-Identified Clusters 1 and 2 in the GSE19234 Dataset**

Figure S2A: The heatmap shows the expression profiles of NK cell exhaustion markers in Clusters 1 and 2. Each row represents a gene, and each column represents a sample. The color scale indicates the expression level, with red representing high expression and green representing low expression. The samples are grouped into two clusters (Cluster 1 and Cluster 2), as indicated by the color bar at the top of the heatmap.

Figure S2B: The boxplot compares the expression levels of NK cell exhaustion markers between Clusters 1 and 2. Each box represents the expression levels for a specific gene in each cluster. The median is indicated by the line within the box, and the whiskers extend to the minimum and maximum values. The significance of the differences between the two clusters is indicated by asterisks (* for p < 0.05, ** for p < 0.01).

Figure S2C: The scatter plots show the correlation between NK cell levels and the expression of NK cell exhaustion markers. Each plot includes a regression line and the coefficient of determination (co.ef) and p-value for the correlation. The x-axis represents the NK cell levels, and the y-axis represents the expression levels of the respective markers.

**Supplementary Figure S3. External Validation of the TIDE Metrics and Their Correlation with Classifier Scores in GSE19234**

Figure S3A: The boxplots show the distribution of TIDE, Dysfunction, and Exclusion scores for Clusters 1 and 2. Each box represents the level of scores, with the median indicated by the line within the box. The whiskers extend to the minimum and maximum values. The significance of the differences between the two clusters is indicated by the Wilcoxon test p-values.

Figure S3B: The scatter plots show the correlation between classifier scores and TIDE metrics. Each plot includes a regression line and the coefficient of determination (co.ef) and p-value for the correlation. The x-axis represents the NK cell levels, and the y-axis represents the respective TIDE metric scores.

**Supplementary Figure S4. External Validation of the Gene Classifier in the GSE65904 dataset**

Figure S4A: The Kaplan-Meier survival curves in Figure S4A show the distant metastasis-free survival (DMFS) and disease-specific survival (DSS) probabilities over time for patients in Clusters 1 and 2. The top panel shows the DMFS curves for Cluster 1 (red) and Cluster 2 (blue). The bottom panel shows the DSS curves for Cluster 1 (red) and Cluster 2 (blue).

Figure S4B: The heatmap shows the expression profiles of NK cell-related genes in Clusters 1 and 2. Each row represents a gene, and each column represents a sample. The color scale indicates the expression level, with red representing high expression and green representing low expression. The samples are grouped into two clusters (Cluster 1 and Cluster 2), as indicated by the color bar at the top of the heatmap.

Figure S4C: The ridge plot and heatmap show the enrichment levels of NK cell-related pathways in Clusters 1 and 2. The ridge plot shows the distribution of pathway scores for different NK cell-related pathways. Each row represents a different pathway, and the curves indicate the distribution of scores within each cluster. The heatmap shows the individual-level of enrichment in these pathways in Clusters 1 and 2. Each row represents a different pathway, and each column represents a patient. Red indicates higher enrichment levels, while green indicates lower enrichment levels.

**Supplementary Figure S5. External Validation of Classifier Scores in GSE244982 for Immunotherapy Response**

Figure S5A: The Sankey diagram illustrates the distribution of classifier scores and their association with response status (responder vs. non-responder) in Clusters 1 and 2. The far-left column represents the range of classifier scores, with each row indicating a specific patient. he middle section shows the distribution of these scores across Cluster 1 (pink) and Cluster 2 (light blue). The far-right column indicates the proportion of responders (red) and non-responders (cyan) within each cluster.

Figure S5B: The bar plot in Figure S5B shows the rates of responders and non-responders in Clusters 1 and 2.

Figure S5C: The ROC curve assesses the classifier's ability to predict responder status, with an area under the curve (AUC) of 0.777.
